# Imaging and Imaging-Guided Interventions in the Diagnosis and Management of Hepatocellular Carcinoma (HCC)-Review of Evidence

**DOI:** 10.5812/iranjradiol.8242

**Published:** 2012-11-20

**Authors:** Hossein Ghanaati, Seyed Moayed Alavian, Ali Jafarian, Nasser Ebrahimi Daryani, Mohsen Nassiri-Toosi, Amir Hossein Jalali, Madjid Shakiba

**Affiliations:** 1 Department of Radiology, Faculty of Medicine, Tehran University of Medical Sciences, Tehran, Iran; 2 Advanced Diagnostic and Interventional Radiology Research Center (ADIR), Tehran University of Medical Sciences, Tehran, Iran; 3 Baqiyatallah Research Center for Gastroenterology and Liver Diseases, Baqiyatallah University of Medical Sciences, Tehran, Iran; 4 Hepatobilliary and Liver Transplantation Division, Faculty of Medicine, Tehran University of Medical Sciences, Tehran, Iran; 5 Department of Gastroenterology, Faculty of Medicine, Tehran University of Medical Sciences, Tehran, Iran

**Keywords:** Carcinoma, Hepatocellular, Radiology, Interventional, Chemoembolization, Therapeutic, Iran, Risk Factors

## Abstract

The imaging of hepatocellular carcinoma (HCC) is challenging and plays a crucial role in the diagnosis and staging of the disease. A variety of imaging modalities, such as ultrasound, computed tomography (CT), magnetic resonance imaging (MRI) and nuclear medicine are currently used in evaluating patients with HCC. Although the best option for the treatment of these cases is hepatic resection or transplantation, only 20% of HCCs are surgically treatable. In those patients who are not eligible for surgical treatment, interventional therapies such as transcatheter arterial chemoembolization (TACE), percutaneous ethanol injection (PEI), radio-frequency ablation (RFA), percutaneous microwave coagulation therapy (PMC), laser ablation or cryoablation, and acetic acid injection are indicated. In this paper, we aimed to review the evidence regarding imaging modalities and therapeutic interventions of HCC.

## 1. Background

Hepatocellular carcinoma (HCC) is the fifth most prevalent cancer worldwide with a poor prognosis and it is the third most common cause of death from cancer (1). It is especially prevalent in Asia where chronic hepatitis B infection is endemic. HCC has generated interest in Europe and the USA due to its increase in the past decade. Chronic hepatitis B virus (HBV) infection is the most common risk factor regarding its endemic presence in the heavily crowded regions such as our country (1, 2). HCC is more prevalent in men and in those who are between 30 and 50 years of age (3). “It was estimated that more than 35% of Iranians have been exposed to HBV and about 2.14% were chronic carriers” (4, 5). Other factors such as metabolic syndrome and diet can affect the occurrence of HCC in the community (6-8).Since surgical resection and local ablation are the most effective therapies for small HCCs, early detection of HCC is critical (9). Imaging is very important in screening, confirming the diagnosis, treatment planning, management and follow up of patients after treatment. In other malignancies of the gastrointestinal and biliary system, the non-invasive approach can help the patients (10). Management of HCC is controversial due to its ever-changing epidemiology, the underlying cirrhosis and the developing in the therapeutic algorithms (11). Surgical resection and liver transplantation are the most effective treatments of HCC; however, less than 20% of HCCs are surgically resectable due to multifocal diseases, proximity to the vital vascular or biliary structures and the insufficient functional hepatic reserve in cirrhosis (12, 13). When surgery is not indicated, interventional radiology procedures such as transarterial chemoembolization (TACE), radiofrequency ablation and microwave ablation are the next steps (14). The aim of this review is to describe the role of imaging and imaging-guided interventions in HCC.

## 2. Imaging of Hepatocellular Carcinoma

**Figure 1:**
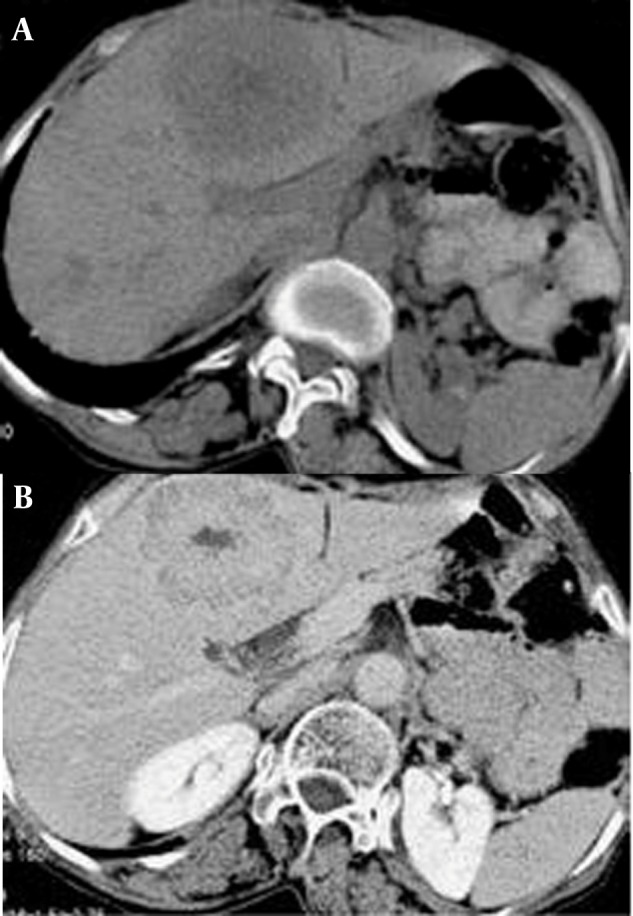
Helical CT in hepatocellular carcinoma A, Hepatocellular carcinoma. Nonenhancing helical CT shows a low density structure (lesion) with an ill-defined border in the left hepatic lobe; B, Hepatic parenchymal phase of helical CT shows a solid enhanced lesion with central scar.

**Figure 2:**
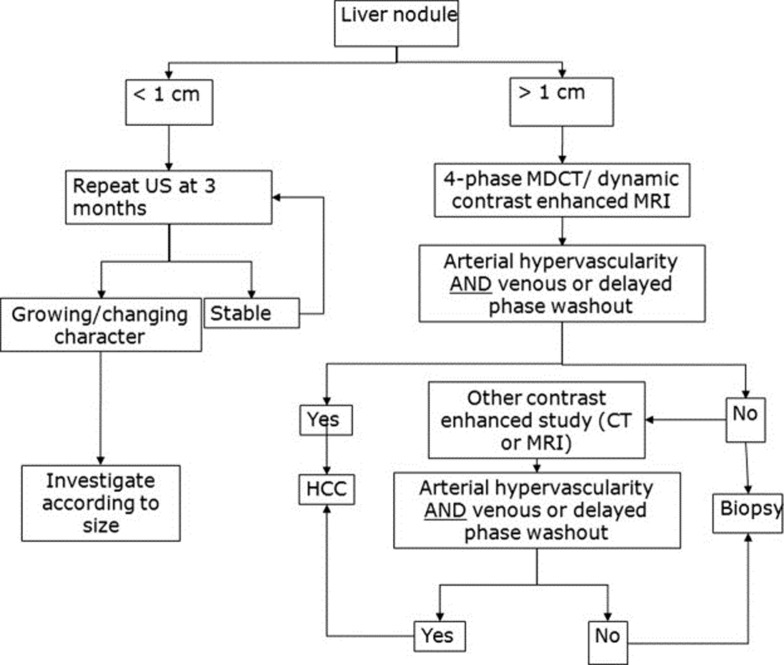
AASLD algorithm for diagnosis of HCC

Detection and characterization of the tumor is the aim of imaging in HCC. The diagnosis is suspected when the lesion shows increased arterial flow, pseudocapsule, internal septa and a mosaic appearance. Generally, HCC has hepatic arterial enhancement. During the portal and venous phases, the tumor fades off and the pseudocapsule shows bright enhancement (15). Larger HCCs are usually heterogeneous and have central necrosis with abnormal internal vessels (16). Ultrasonography, CT scan and MRI are diagnostic modalities which are used in the diagnosis and follow-up of HCC (Figure 1). The American Association for the Study of Liver Diseases (AASLD) algorithm for diagnosis of HCC is shown in Figure 2 (16).

The best imaging method should be sensitive in the detection of hypervascular lesions and it must be able to differentiate arterio-portal shunts from true lesions. It must be able to distinguish pseudocapsule, internal septa and mosaic appearance. Dynamic contrast-enhanced MR is the best imaging method for these reasons (17-20).

### 2.1. Ultrasound

Ultrasonography (US) is the first screening and widely accepted method in the depiction of hepatic normal tissue and lesions due to its safety, quick performance and cost-effectiveness (19). However, it has a sub-optimal sensitivity and specificity in cirrhotic patients, and contrast-enhanced CT or MRI is indicated in high-risk suspicious cases (21). Most HCC lesions are hypo-echoic and have affinity to spread to the portal and hepatic veins as well as biliary ducts and these cause thrombosis and jaundice (9). Based on the European Association for the Study of the Liver (EASL) and AASLD, ultrasonography is the advised method for observation of patients at risk of HCC (6). In general, the mean doubling time of HCC tumors is six months which makes six months a reasonable screening time interval for HCC with ultrasonography (22-24). Sherman et al. employed ultrasonography and serum alpha fetoprotein (AFP) in a group of non-cirrhotic hepatitis B carriers and found that ultrasonography had a sensitivity of 78.8% and specificity of 93.8% (25). Differentiation of small lesions in cirrhotic patients and vascular volume evaluation could be improved by contrast-enhanced sonography (16, 26). One drawback of contrast-enhanced sonography is that the images can be acquired when rupture of the microbubbles have not happened and imaging of this needs precise planning of the imaging time and image plane position. Ultrasound elastography is a new technique that uses strain as a parameter and shows mechanical aspects of tissue stiffness after compression of the probe on tissues such as the breast. Due to the location of the liver (deep and surrounded by ribs and lungs), its compression is impossible and using ultrasound elastography is not possible, unless acoustic radiation force impulse elastography with a short, high-intensity, focused US beam is used for tissue displacement (27). Tissue displacement in a region of interest to the acoustic radiation force depends on the tissue’s viscoelastic characteristics and is inversely proportional to tissue stiffness. Although elastography can assess the stiffness of superficial tissues, acoustic radiation force impulse elastography can estimate the stiffness of the superficial and deep tissues due to application of shear wave (9). When applied with conventional ultrasonography, this technique has some advantages in differentiation between malignant and benign lesions as well as improving conspicuity of the lesions with unclear margins on the B-mode image in focal liver masses (7).

### 2.2. Computed Tomography (CT)

Triphasic and quadriphasic (pre, arterial, portal and equilibrium) dynamic CT images are useful for the evaluation of focal liver lesions diagnosed on sonography and to assess patients who have elevated levels of α-fetoprotein with normal sonography. CT-scan is also applied for the staging of HCC as well as follow-up of patients after surgical resection, radiofrequency ablation or percutaneous ethanol injection. Regenerative nodules are visible on unenhanced CT in 25% of the patients as hyperattenuating nodules (28, 29). These nodules are typically iso-intense on contrast-enhanced CT and are not noticeable from the surrounding liver parenchyma and arterial hypervascularity is the hallmark of HCC. Multi-Detector CT (MDCT) has some advantages such as a shorter scan time, thinner sections and a longer scan range. Therefore, double arterial phase images and isotropic volume imaging is practical with MDCT for hypervascular HCC.Perfusion CT using MDCT is the technology which offers a wide range of clinical and research applications in the fields of oncology and stroke. It is used for diagnosis, staging and evaluation of treatment response and prognosis of liver tumors (9). Perfusion CT is based on temporal changes in tissue attenuation after injection of iodinated contrast materials (9). Protocols of perfusion CT that are used for analysis according to the physiologic parameters include a first-pass study, a delayed study or both. The first-pass study consists of images obtained in the first cine phases for about 40-60 seconds. To obtain permeability measurement, the second phase for permeability assessment ranges from 2 to 10 minutes and is after the first-pass examination. A contrast media with the bolus injection (40-70ml) in the rate of 3.5-10 ml/sec is necessary for optimal perfusion assessment (30). There is an increasing trend in the application of perfusion CT in the field of oncology including lesion characterization, diagnosis of occult malignancies, provision of prognosis and monitoring the efficacy of treatment (30). Malignant hepatic malignancies show high perfusion values in comparison with the normal tissue (31, 32). In terms of treatment evaluation, HCC shows a fall in blood flow, blood volume and permeability after antiangiogenic therapies or trans-arterial chemoembolization (33, 34). The hallmark of HCC on contrast-enhanced CT and MRI is arterial hypervascularity with washout of intra-lesional contrast on portal, venous and delayed phase images (17). The dynamic-enhanced acquisitions after the injection of extracellular contrast agents may help the depiction and characterization of HCC. The extracellular contrast materials distribute from the intravascular to the interstitial space. A typical enhancement is described as an early arterial uptake followed by washout in the porto-venous or delayed phases. 

### 2.3. Magnetic Resonance Imaging (MRI)

**Figure 3:**
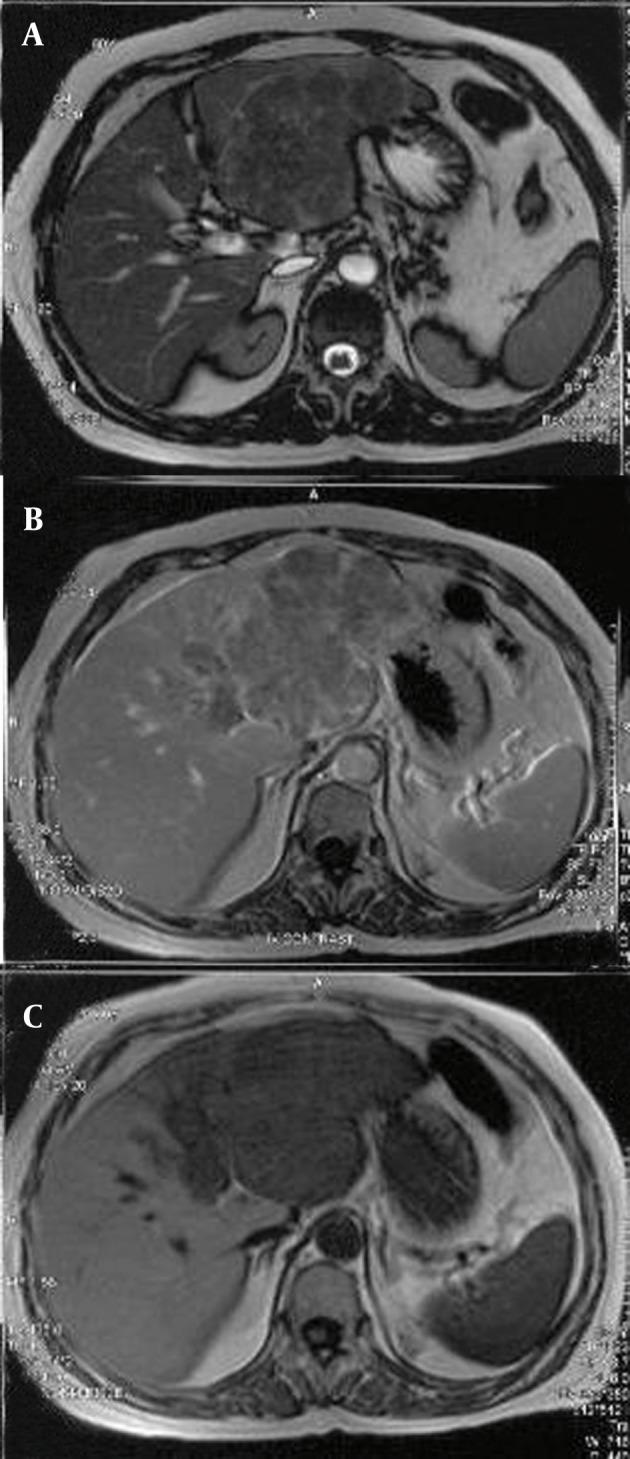
MRI in HCC A, Axial T2-W image shows heterogeneous high signal area, pseudocapsule and central hyposignal area in favor of necrosis; B, T1-W image after gadolinium injection shows enhancement of the mentioned mass in the left lobe; C, Axial T1-W shows hyposignal mass in the left lobe.

MRI should be performed in patients who have non-diagnostic findings on CT or patients in whom iodinated contrast agents are contraindicated (Figure 3).MRI is superior to CT in T2 hyperintense malignant lesions and the sensitivity of dynamic contrast-enhanced MRI is more than dynamic contrast-enhanced CT (84% vs. 47%); also MRI can depict smaller lesions (1-2 cm in diameter) in comparison with CT (20). MR shows better efficacy in comparison to CT on decision making for the management of patients (90% vs. 77-80% for decision making) (35, 36). HCC patients show variable signal intensity on T1-weighted images and most of them have mild hyperintense signals on T2-weighted sequences. Contrast enhancement is useful in the detection of most HCCs in the arterial phase and well-timed arterial phase imaging is essential for depiction and characterization of HCCs during dynamic MRI. MRI is also beneficial in T2 hypointense nodules smaller than 1 cm in size and also T2 hypointense dysplastic nodules greater than 1 cm (37). Although regenerative nodules have variable signal intensity on T1 weighted images, they are usually iso-intense in this sequence; while on T2-weighted images, they have low signal intensity.The use of contrast agents such as super paramagnetic iron oxide particles and gadobenate dimeglumine may increase the sensitivity and specificity of MRI in the detection of HCC (38). For those patients who are undergoing staging studies using liver specific MRI contrast agents, a dynamic scan with gadolinium chelates can be done immediately afterwards with a second injection, which results in a dual contrast MRI study (39). Diffusion-weighted image (DWI) is a non-invasive quantification of water diffusion and microcapillary blood perfusion without gadolinium contrast material, which is especially important in those cases with renal dysfunction at risk for nephrogenic systemic fibrosis (40). Using DWI we can increase the detection rate of focal liver lesions and HCC with gadolinium-enhanced MR in cirrhotic patients (41). Another application of DWI is treatment monitoring after chemotherapy or chemoembolization in HCC and other liver tumors (42). Cellular necrosis causes increased membranous permeability and subsequently free diffusion of water molecules and finally increase in the ADC value that enables early detection of cellular necrosis (42). Magnetic resonance elastography (MRE) is a non-invasive quantitative technique for evaluation of mechanical properties of the tissue. This technique is useful in quantitative tissue characterization for differentiating benign and malignant liver tumors (9).

## 3. Pathological Diagnosis of HCC

The gold standard modality in the detection of HCC is cytological examination of a suspected lesion, which is achieved by biopsy. Computed tomography (CT) has been applied as the guiding imaging modality for interventional procedures and CT fluoroscopy (FCT) is a new technique which causes real time reconstruction of the images. This technique produces faster image reconstruction, near continuous image update and convenient in-room table control and image viewing during CT-guided procedures. In our study, the success rate of FCT (92%) was significantly higher than conventional CT for taking liver mass specimens (92% vs. 65%) (P = 0.019) (43).

In hemophilia patients who were exposed to blood-borne hepatitis viruses after treatment with clotting factors, liver biopsy is necessary for assessment of chronic liver disease and determination of the necessity of interferon therapy. Transjugular liver biopsy (TJLB) is a procedure which should be performed by an interventional radiologist. In our experience on 12 HCV patients with congenital bleeding disorders (CBD) and elevated liver enzymes, we performed TJLB with a 100% success rate in access to the hepatic veins and a 92% success rate in tissue obtaining. In four patients (36.4%) we had mild hepatitis and in five cases (45.4%) we had moderate hepatitis. Extensive fibrosis or cirrhosis occurred in two patients (18.2%) and we had two procedure-related complications (16.6%) (44).

## 4. Interventional Procedures in the Treatment of HCC 

Interventional procedures in the treatment of HCC are divided into two groups of non-vascular (chemical and thermal ablations) and vascular interventions. Among chemical ablations, ethanol injection and acetic acid injection could be mentioned and some examples of thermal ablations are radiofrequency ablation (RFA), microwave ablation, laser ablation and cryoablation.

### 4.1. Percutaneous Ethanol Injection

The first interventional therapy for HCC was percutaneous ethanol injection (PEI), which causes dehydration of proteins, coagulation necrosis, cell fibrosis and ischemia of neoplastic tissues and is applied in tumors which are not in advanced stages (45). PEI is performed with local anesthesia using ethanol 95% via a 21-gauge needle guided by ultrasound under local anesthesia. This procedure should be performed in four to six sessions once or twice weekly based on the tumor size, pattern of tumor perfusion and the patient’s compliance. No remarkable damage to the remaining parenchyma, less complications, low cost, easy repetition in new lesions, easy operation and also good long-term results are some advantages of this procedure (28, 46-48). Complications of this procedure are hepatic failure, abscess, intra peritoneal hemorrhage, biloma and cholangitis. (47). Existence of gross ascites, bleeding and obstructive jaundice are among contraindications of PEI (3, 47). In HCCs smaller than 3 cm in size, there is complete response in 90% to 100% of the cases and in 3-5 cm and larger than 5 cm in diameter tumors, the complete response rate is 70% and 50%, respectively (49). In one study which was performed by Livraghi et al., the 3 and 5-year survival were 79% and 47%, respectively for patients with Child class A who had a single lesion smaller than 5cm (50). One, two and three-year survival rates after PEI, radiofrequency ablation (RFA) and microwave ablation (MWA) are shown in Table 1.

**Table 1:** Comparison Between Survival Rates of Percutaneous Ethanol Injection (PEI), Radiofrequency Ablation (RFA) and Microwave Ablation

Method	Number of Patients	1-Year Survival, %	2-Year Survival, %	3-Year Survival, %
**Percutaneous Ethanol Injection (51) **	162	90	80	63
**Radiofrequency Ablation (52) **	52	100	98	-
**Microwave Ablation (53) **	50	96	83	73

### 4.2. Acetic Acid Injection

Acetic acid injection is a minimally invasive procedure with tissue diffusion and a necrotizing power better than ethanol (48, 54). Although in a prospective randomized clinical trial study the authors concluded that cancer-free survival and overall survival rates were higher in acetic acid group in comparison with ethanol injection group (54), two other papers reported similar outcomes after acetic acid and ethanol injection in the treatment of HCC (21, 54, 55). Although in a study carried out by Germani et al., the sample size was small, acetic acid injection did not differ significantly from PEI, considering both HCCs smaller and greater than 2 cm in diameter (56). Fever is a side effect of this procedure, which may be due to tumor necrosis and decreased immunity of the patients. A prophylactic antibiotic therapy is necessary before treatment in high risk cases to avoid bacteremia and infection complications. 

### 4.3. Radiofrequency Ablation

**Figure 4:**
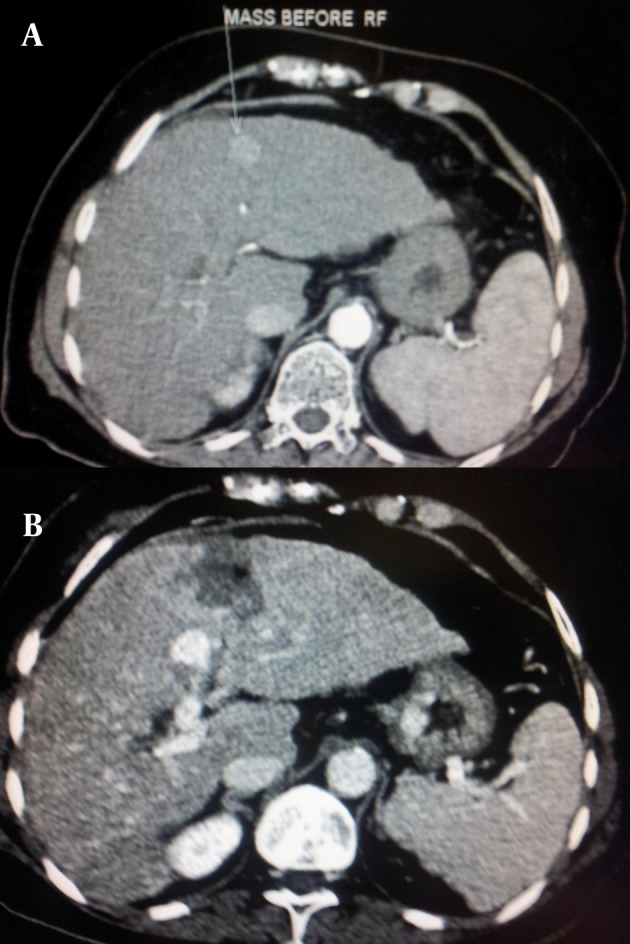
A, Dynamic CT scan, arterial phase shows hyperdense high vascular mass in the 4th segment of the liver; B, Dynamic CT scan, arterial phase, 5 min after RFA, shows a hypovascular area and complete vanishing of the enhancement.

Coagulative necrosis of the tumor using electrical heating around a probe generating electromagnetic radiation occurs after RFA (Figure 4) (57-59).

 MRI, CT-scan or ultrasonography are imaging modalities which are used for probe guidance. Contraindications of RFA for HCC are a platelet count less than 50000, hemostasis disturbances, refractory ascites, jaundice and having pacemakers. Relative contraindications are lesions near the gastrointestinal tract, billiary system and the heart (60). For central lesions near the hilum there is a risk of vascular or biliary tract injury and for tumors which are located within 1cm of the hepatic portal tract, RFA should be avoided.

The efficiency of RFA mostly depends on the size and location of the tumor. For those tumors greater than 3 cm, the efficacy of RFA decreases with the increase in size and complete response may occur in lesions with 3-5 cm diameter (57, 61). The major complications of RFA are liver failure, hemorrhage, infection, abscess, intercostal nerve injury, adjacent organ injury, tumor lysis syndrome and pneumothorax (45, 57). In a randomized trial of RF ablation versus surgical resection in patients with a solitary HCC less than 5 cm in diameter, no differences in the overall survival rates and cumulative recurrence-free survival rates were observed (62). In another randomized clinical study, Shiina et al. (63) compared PEI and RFA in 114 and 118 patients with early stage HCC, respectively and they reported 100% complete response after both procedures. The two and three-year survival rates were 82% and 63%, respectively for PEI group and 90% and 80%, respectively for RFA group.

### 4.4. Microwave Ablation (MWA)

Microwave ablation is a thermal ablation therapy in which there is a coagulation area that provides reliable ablation for HCC (64). Microwave ablation is used chiefly for tumors less than 2 cm in size, but for larger tumors, the efficacy of this procedure is less acceptable. It is better to apply multiple electrode insertion for a better ablation (53). Two microwave ablation sessions are accepted in each nodule (53) and the second session is more efficient than the first due to destroying of blood vessels in the first session which provides a more effective situation for the second session. A microwave coagulator produces and transmits energy into a monopolar needle electrode that is introduced into the tumor. Molecular vibration results in the increase of tissue temperature and subsequently thermal coagulation around the electrode, which is placed via a 14-gauge needle that is inserted into the tumor under imaging (CT or ultrasound) guidance (65).

WHO performance stages of 3 or The reported 1-, 2-, 3-, 4- and 5-year survival rate of patients with HCC (mean diameter 4.1 ± 1.9 cm) that have been treated by this technique are 92.7%, 81.6%, 72.9%, 66.4%, and 56.7%, respectively (65). Dynamic contrast - enhanced CT is useful for evaluation of microwave ablation and is usually performed 7-14 days after the procedure (53).

### 4.5. Laser Ablation

Laser ablation is a safe and effective procedure in patients with a small unresectable HCC (5 cm or less) restricted to the liver with a low liver reserve or disturbed liver function. This method causes thermal destruction of the tissue by conversion of absorbed light into heat (66). Contraindications of laser include gross ascites, uncorrectable coagulopathy and obstructive jaundice. In tumors which are located under the diaphragm or are too near to vital structures such as the bile ducts, major blood vessels and the stomach, the procedure can be difficult with higher complications (67). A study performed by Pacella et al., in a group of 169 lesions in 148 patients, the authors reported long term survival rates of 89%, 52% and 27% after 1, 3 and 5 years, respectively (68). Pleural effusion, post procedural fever, severe pain and tumor seeding are complications of laser ablation.

### 4.6. Percutaneous Cryoablation

In cryoablation, we apply -196°C temperature to the tumor by a probe, which is located in the tumor and cooled using circulating liquid nitrogen or argon. As the tissues around the lesion remain intact, we can apply this method safely for multiple lesions, and those cases with more than three unresectable tumors less than 5 mm (69). This method is very difficult when the nodules are 6 cm or more in diameter and patients in whom more than half of the liver is involved have higher morbidity (70). Hypothermia, hemorrhage, bile fistula and collections, cryoshock, biliary leak or subsequent stenosis, liver capsular cracking, asymptomatic right-sided pleural effusion, liver abscess and transient thrombocytopenia are among complications of this procedure (67). In a study conducted on 235 patients for evaluation of the survival after cryoablation, Zhou and his colleague reported a 39.8% 5-year survival and for their 80 patients who had lesions smaller than 5 cm, this figure was 55.4% (69). In another study, Crews et al. performed cryoablation for 40 patients with hepatic tumors and their 18-month survival rates for HCC was 60% (71).

### 4.7. Transarterial Chemoembolization (TACE)

Since the main blood supply of HCCs greater than 2 cm is provided by the arterial system, transarterial chemoembolization (TACE) that includes both arterial blockage making ischemia and local chemotherapy has been approved as the gold standard treatment of unresectable HCC in preserved liver function patients without extrahepatic tumor spread (72). TACE includes local injection of two combined components; namely, embolization and chemotherapy agents. The embolization is done mainly by lipiodol injection in the tumor-supplying artery (an oily contrast media with selective accumulation in the tumor) (73). The chemotherapeutic agent is mainly doxorubicin or cisplatin (73). According to the guidelines of AASLD and EASL, TACE is the non-curative, ﬁrst-line therapy for advanced-stage tumors in patients with large or multifocal HCC without vascular invasion or extrahepatic spread who are not candidates for surgery (74). Contraindications include severely impaired liver function (Child-Pugh C), a serum bilirubin level higher than 2 mg/dl, clinically relevant refractory ascites, coagulopathy, signiﬁcant thrombocytopenia, encephalopathy, active gastrointestinal bleeding, signiﬁcant comorbidity (cardiac and/or renal failure), a tumor burden greater than 50% or end-stage tumorous disease (Okuda III), poor performance status (Karnofsky index < 70%) or WHO performance stages of 3 or 4, extrahepatic metastases, vascular invasion, portal vein occlusion due to thrombosis or liver tumor, hepatofugal blood ﬂow in the portal vein or patients with a transjugular intrahepatic portosystemic shunt. In fact, TACE could be considered in patients contraindicated for surgery with early-stage disease. In addition, TACE is an option in a cirrhotic patient candidated for transplant (67, 73, 74).

### 4.7.1. Preprocedure Workup

The tumor will be assessed by contrast-enhanced MRI (including perfusion and diffusion sequences); if not feasible, dual-phase MRI or CT will be performed. In addition to delineation of the tumor extent and necrosis, this imaging provides detailed information of the anatomy of the celiac trunk and its branches and portal vein invasion or thrombosis (51). Since the rate of liver abscess is lower than 1%, prophylactic antibiotic before TACE is controversial (52, 66). Regarding the borderline conditions and critical situation of the patients, many prefer to use antibiotics for coverage against enteric gram negative bacteria and exogenous gram positive bacteria entering during the procedure (75). As some baseline conditions such as poor liver function and especially previous biliary surgeries (speciﬁcally previous whipple surgery) increase the risk of liver abscess, the routine use of prophylactic antibiotics has been recommended in these situations (73, 75).

### 4.7.2. Procedure

**Figure 5:**
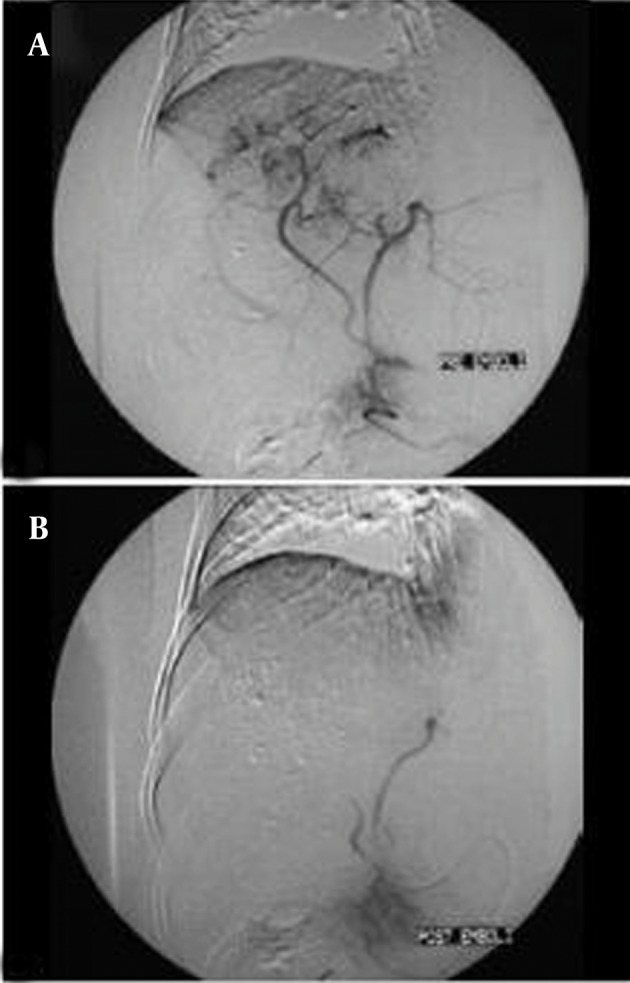
A, Neovascularity and hypervascular lesions in segments 7 and 8 of the liver; B, Complete obliteration of vascular structures within the tumor is seen. Lipiodol droplets are also visualized in the texture of the tumor.

First of all, for selection of the best vessel branches for TACE, a diagnostic angiography of the celiac trunk and superior mesenteric artery with late-phase imaging of the portal venous system will be done. This is performed via insertion of a 4F introducer sheath in the right common femoral artery (72, 74) for determining the arterial supply to the tumor, detecting possible variations in the hepatic arterial system, identifying the arteries that should be avoided during treatment and determining the patency of the portal vein or the presence of hepatopetal ﬂow through collaterals to the liver in case of portal vein tumor thrombosis. Selective or super selective catheterization with the use of a large-hole microcatheter is preferred. Using a 4F hydrophilic cobra catheter with a hydrophilic guide-wire is enough in about half the cases. The catheter should not be less than twice the diameter of the vessel. In small vessels, the only route for access is using microcatheters designed for TACE. Coil-embolization of all distal ends of non-hepatic artery branches distal to the microcatheter tip is recommended. An arteriogram before injecting any chemotherapic agent is recommended. Complete blockage of the tumor-feeding branches is the goal of procedure, thus it is essential to check for extrahepatic collateral arteries feeding the HCC. An exophytic tumor growth or subcapsular location or peripheral iodized oil retention defect within the tumor or a peripherally located portion of viable tumor on a follow-up CT scan are in favor of external collateral artery (ExCA) feeding the tumor (Figure 5).There are some controversies about how selectively the catheter tip should be placed (lobar or segmental) during TACE (76). Some authors believe that the selective TACE is better than non-selective therapy as the selective method maximizes the drug impact on the tumor and concurrently lowers the damage to normal liver tissues. Thus, they recommend advancement of the catheter tip to the tumor as close as possible in the feeding artery (segmental or subsegmental)(77, 78).The chemoembolization agent is prepared as an emulsion that consists of up to 20 mL of lipiodol and 10-50 mg of doxorubicin hydrochloride; the dose is adjusted according to the size, extent and vascularity of the tumor (78).The right and left hepatic arteries should still be patent at the end of injection, while the ﬂow in the second and third-order branches should be reduced and tumor blush should not be seen. After catheter removal, a plain abdominal ﬁlm or cone beam CT of the hepatic region is performed to assess the focal uptake of lipiodol into the HCC nodules. Two to four TACE sessions are required depending on the arterial anatomy to treat the entire liver (the second procedure should be performed after 3-4 weeks) The response will be evaluated by repeated imaging studies and tumor markers (51, 67). The sequential TACE is safe in many patients and could increase the survival of the patients (79). In addition to routine agents, a new microsphere that can serve as a drug eluting agent has been introduced in the recent years. It is named DC-Beads and it could provide a precisely controlled and sustained release of chemotherapeutic drugs. It seems that the type of embolic agent does not inﬂuence the overall survival (72, 73, 80).

### 4.7.3. Complications

The main complication that occurs in many of the patients is the postembolization syndrome (PES) that has variable manifestations. Typical symptoms include abdominal discomfort, pain, nausea, vomiting, fatigue and fever, which may last for a few hours up to 5 days. Probably it is due to tissue ischemia and an inﬂammatory response to chemoembolization. Although PES is the major cause of postprocedural hospitalization and future compliance, it could be managed symptomatically. Hepatic failure and hepatorenal syndrome are two life-threatening complications that are more likely in patients with reduced liver function and/or reversed or occluded portal vein ﬂow. Fortunately, impaired liver function is reversible in the majority of patients. Other complications include liver abscess (in the necrotic liver tissue) and septicemia. Ischemic cholecystitis, pancreatitis and gastric erosions or ulcers are other complications due to inadvertently injection of agents into the organs. Bile duct injuries are uncommon. Rare complications include embolic events in the gastrointestinal tract, pulmonary or cerebral circulation due to lipiodol (72, 73).

### 4.7.4. Efficacy

Many systematic reviews have shown the prolongation of survival after TACE in unresectable HCCs. Important aspects of all such treatment modalities are their effective tumor responses, minimal toxicity and sparing of the normal liver tissue (53, 81, 82). Llado et al. reported the overall actuarial survival rates of 61%, 32% and 19% at 12 months, 24 months and 36 months, respectively (83). O’Suilleabhain studied 320 patients who underwent TACE and reported the median survival from the initial TACE treatment was 72.3 months. They had three patients diagnosed as disease-free at the end of the study. They reported the unilobar tumor, an albumin concentration greater than 35 g/l and α-fetoprotein level below 1000 ng/ml as independent prognostic factors for survival (84). 

## 5. Combination of Interventional Therapies 

### 5.1. PEI and RFA

One advantage of PEI is its efficacy in the treatment of residual or recurrent tumors after RFA or cryoablation. Due to anatomical problems such as proximity to vessels, which cause incomplete ablation and difficulties to destroy, PEI can be a useful modality (85). In those patients who have tumors near the dome of the liver and are not candidates for laparoscopic or surgical treatment, chemical ablation may be considered as the first-line therapy (85).

### 5.2. PEI and TACE

For those tumors with more than 3 cm diameter, efficacy of RFA after TACE may be increased as a result of pathological changes after TACE, which increase the diffusion of ethanol through the tumor (86). In one randomized clinical trial study on 30 patients with less than four HCCs smaller than 3 cm, who underwent PEI or TACE and PEI; the necessity for additional PEI sessions was less in the combination therapy group (mean; 3.8 sessions versus 5.3 sessions) (86). The authors also reported a higher local recurrence rate in those who underwent PEI in comparison with the combination therapy group after two years (65% vs. 35%). The 3-year survival rate for tumors with less than 2 cm diameter after PEI alone and in combination therapy was 62% and 100%, respectively (86). In another randomized trial study, the authors concluded that the survival rate in the group with a single session of combined TACE and PEI was similar to that with two to five sessions of TACE alone in HCCs with 3-8 cm size in diameter (87).

### 5.3. RFA and TACE

A combination therapy which consists of RFA and TACE may have a synergistic effect in the treatment of HCC, especially in those large lesions that do not respond to each procedure alone. Reduction in blood flow in the tumor after TACE causes decrease in the heat sink phenomenon, which could limit the ablation area (88, 89). TACE before RFA shows increase in the RFA zone and the probability of a complete response, especially in nodules larger than 3 cm (88, 89). Complete response rate of RFA after TACE for tumors with 5-cm diameter is about 90%-100% at 1 year, which is similar to the response to RFA alone for the lesions with 3-cm diameter (23, 89). The risk of mortality and complications after combination therapy (RFA after TACE) is about 4% which did not show a significant difference with RFA alone (23).

### 5.4. MWA and TACE

TACE and MWA are complementary and their combination is preferred especially for large HCCs. Moreover, their combination may cause decrease in the number of microwave antenna and irradiation time. Similar to other thermal ablation therapies, the coagulation diameters for MWA may be influenced by the cooling effect and interruption of blood flow can increase the coagulation diameters (90, 91). TACE in combination with MWA may cause increase in the ablation volume and thereafter, MWA can destroy the peripheral zone of the viable tumor after TACE. In one study on 18 patients with HCCs (more than 2 cm, but less or equal to 3 cm in diameter), the authors performed MWA 1-2 days after TACE and found that tumor necrosis occurred in 17 patients (92).

### 5.5. Laser Ablation and TACE

The combination of laser with TACE is effective, especially for large lesions. In one study conducted by Pacella et al., the authors applied laser followed by TACE in the treatment of 30 large HCCs (3.5-9.6 cm) and achieved a 90% ablation rate with survival rates of 92%, 68% and 40% at 1, 2, and 3 years, respectively with no significant complications (93). In another study, the authors supported the results of the study performed by Pacella et al. for improvement in the ablation rate as well as survival in patients with HCCs larger than 5 cm (94).

## 6. Conclusions

In conclusion, imaging (mainly CT and MR imaging) has a crucial role in the diagnosis and assessment of tumor response in patients with HCC and interventional therapies play a crucial role in the management of those who are not candidates for surgical therapies.
